# The economic cost of Alzheimer's disease: Family or public health burden?

**DOI:** 10.1590/S1980-57642010DN40400003

**Published:** 2010

**Authors:** Diego M. Castro, Carol Dillon, Gerardo Machnicki, Ricardo F. Allegri

**Affiliations:** 1 MD, Servicios de Neuropsicología (SIREN) y Neurología, Instituto Universitario CEMIC, Buenos Aires, Argentina.; 2 MD, Laboratorio de Memoria, Servicio de Neurología, Hospital General Abel Zubizarreta, Buenos Aires, Argentina.; 3 MSc, Laboratorio de Memoria, Servicio de Neurología, Hospital General Abel Zubizarreta, Buenos Aires, Argentina.; 4 MD and PhD, Servicios de Neuropsicología (SIREN) y Neurología, Instituto Universitario CEMIC, y Laboratorio de Memoria, Servicio de Neurología, Hospital General Abel Zubizarreta, Buenos Aires, Argentina.

**Keywords:** Alzheimer’s disease, economic costs, health economics

## Abstract

Alzheimer’s disease (AD) patients suffer progressive cognitive, behavioral and functional impairment which result in a heavy burden to patients, families, and the public-health system. AD entails both direct and indirect costs. Indirect costs (such as loss or reduction of income by the patient or family members) are the most important costs in early and community-dwelling AD patients. Direct costs (such as medical treatment or social services) increase when the disorder progresses, and the patient is institutionalized or a formal caregiver is required. Drug therapies represent an increase in direct cost but can reduce some other direct or indirect costs involved. Several studies have projected overall savings to society when using drug therapies and all relevant cost are considered, where results depend on specific patient and care setting characteristics. Dementia should be the focus of analysis when public health policies are being devised. South American countries should strengthen their policy and planning capabilities by gathering more local evidence about the burden of AD and how it can be shaped by treatment options.

## Abstract (pt)

O paciente com doença de Alzheimer (DA) sofre comprometimento progressivo cognitivo, comportamental e funcional, que resulta numa grande sobrecarga aos pacientes, familiares e à saúde pública. A DA inclui custos diretos e indiretos. Os custos indiretos (como perda ou redução dos ganhos pelo paciente ou membros da família) são os mais importantes custos dos pacientes leves e na comunidade. Os custos diretos (tais como tratamento médico ou serviços sociais) aumentam com a progressão da doença, quando o paciente é institucionalizado ou quando um cuidador formal é requerido. A terapia com drogas representam um aumento nos custos diretos, mas podem reduzir alguns outros custos diretos ou indiretos envolvidos. Vários estudos projetam uma economia global da sociedade quando é usada terapia com drogas e todos os custos relevantes são considerados; e os resultados dependerão de um paciente específico e características do meio envolvido no cuidado. A demência pode ser um assunto de análise quando as políticas de saúde são desenhadas. Os países da América do Sul deveriam fortalecer suas políticas e capacidades de planejamento, pela geração de maiores evidências locais sobre a sobrecarga da DA e como poderia ser norteada pelas opções de tratamento.

Alzheimer’s disease (AD), the most frequent form of dementia, is a progressive, degenerative brain disease characterized by impairment of cognitive, behavioral and functional abilities that principally affects the elderly. AD is a clear example of an age-related disease. The disorder slowly progresses to a loss of functional abilities and finally to complete dependency. Dementias and AD have become a focus of attention in clinical practice and medical research given the disproportionate rise in the number of elderly people (65 years and older). The social, economic and health care impact is enormous. Economic costs of AD are significant for the health system given the resources deployed to prevent, diagnose, treat and manage dementia. The costs of dementia to society extend beyond these direct costs, as the disease impacts individuals, families and careers both economically and in terms of their quality of life. This review explores the economic impact of AD on health systems and society.

## Epidemiology

Dementias, and particularly AD, are closely linked to ageing. In AD, the prevalence rate doubles every 5 years and various epidemiological studies have shown the exponential growth of prevalence rate with age, starting from around 1.5-2.5% in the 65-69-year bracket, reaching almost 40% in the 90-94 year age group.^1,2^ In addition, according to the World Health Organization (WHO) the proportion of people over 65 years is set to increase from 6.8% in 2000 to 16.2% by 2050.^3^ As a consequence, the prevalence of dementia in 2005 (24 million) is expected to rise to 81 million by 2040, assuming no changes in incidence, mortality and current preventive or curative treatments for these disorders.^4^ The epidemiology of AD gives reason to expect an enormous growth in the social and economic impact of dementias in developed and developing countries.

## Cost definitions

Before reviewing the cost of AD, cost terminology must first be defined. The costs of dementia to society are the result of all goods and services that are given up to prevent, diagnose, treat and otherwise cope with dementia. Individuals, families and carers are affected both economically and in terms of quality of life.^5^ Total costs are divided into direct costs (represented by hospital resources, medical services, drugs, social services, family payments to formal caregivers) and indirect costs (such as loss of income by the patient and loss or reduction for family members or careers). Finally, some literature defines intangible costs as those related to pain or deterioration of patient and caregivers’ quality of life. Only direct and indirect costs are estimated in most studies. Intangible costs are perhaps best defined and captured by health-related quality of life studies and are not covered in this review.

## Economic considerations

### Global burden of illness and overall dynamic of costs in AD

After cancer and coronary heart disease, AD is the third most expensive disorder in the United States,^6^ and with the aging of society will likely become even more significant. Total worldwide societal costs were estimated to be US$ 315 million in 2005^7^ with about 70% of costs occurring in developed countries.

Many studies have evaluated the costs of AD^5,8-13^ and some studies have focused on the relationship between severity and costs.^5,8,11,14-16^ Others have focused on the impact of drug therapies on the cost of dementia,^17-20^ neuropsychiatric disorders,^21^ functional and dependency grade of patients^22^ and co-morbid medical conditions.^23^

The median survival of patients with AD is 9 years for persons diagnosed at age 65 years and 3 years for persons diagnosed at 90 years of age,^24^ and if patients do not die of other causes the disease is expected to progress to advanced stages. Global social costs of AD are dependent on severity of illness, on stage of disease and patient’s place of residence. In mild and moderate AD (early in the disease) the indirect costs are considerable and often exceed direct costs because most patients have an informal caregiver and are living in the community. Thus, the distribution of costs for community-dwelling patients is 40% for direct and 60% for indirect costs.^5,11^ This situation does not represent direct monetary expenditure but a heavy burden on caregivers. If this informal caregiver time is not available, caregiving must be provided by paid carers or supplied through patient institutionalization (two ways of converting indirect into direct costs).

Institutionalization in AD patients is proportional to severity: up to 62% of patients at a severe stage (MMSE <11) living at nursing home against 20% in moderate AD (MMSE 12-16).^5^ When patients are institutionalized, costs shift from indirect to direct. Fifty to 75 percent of total cost of AD occurs during severe stages, principally generated from nursing homes expenses which represent the main expenditure in this illness.^5,25^ The impact of institutionalization cost is significant: annual total cost in Argentina was calculated at US$ 8129.7 and US$ 14863.6 for community-dwelling and institutionalized AD patients, respectively.^5^

Impact of expenses of dementia on the family budget is sufficiently high to account for 66-75% of household income.^47^

## Indirect costs in caring for AD

Because of the nature of AD, progressive impairment in cognitive, behavioural and functional status generates a significant burden on patient care. Informal caregiver time represents an important resource which increases with AD stage in community-dwelling patients. Studies show that informal caregiving time for community-dwelling patients with AD ranged from 11 to 70 hours per week.^40^ This time reduces for institutionalized patients. If this informal caregiver time were not available or becomes too burdensome, use of paid caregivers or institutionalization would be the consequence, with corresponding increases in direct costs.^5^

About 80% of AD patients live at home and receive informal care^42^ while half of all non-institutionalized patients have more than one caregiver. Primary carers are predominantly women, either wife or daughter,^5,12^ and cultural differences can be important in this issue. General carers provide companionship, assist the patient in most non-instrumental or basic activities (eating, dressing, habitual cleanness generally, etc.) and instrumental activities (phone use, cooking, finance management, transport, drug provision, etc.) of daily living. These carer responsibilities increase as dementia progresses, at least until the point at which the person with dementia requires formal care (institutionalization or formal paid caregiver).^41^ Zhu et al.,^42^ reported that informal and formal care (home health service) may coexist. Their study showed that home health and informal care utilization related differently to patients’ clinical characteristics: higher utilization of home health services was significantly associated with function, depressive symptoms, being female, and not living with a spouse; higher utilization of informal care was significantly associated with cognition, function, comorbidities, and living either with a spouse or a child.

Due to the large amount of time spent on patient care, the impact on the health of caregivers is an important issue to consider. Prevalence of depression in caregivers is as high as 30%, and more frequent in female caregivers.^43,44^ Excessive burden is noted by families of patients according to higher frequency of behavioural disturbances, urinary and faecal incontinence and greater need for personal care. Anxiety levels are higher in dementia caregivers than controls and this may be associated with poorer physical health of carers of people with dementia.^45,46^ Other authors suggest that high costs could be considered an indicator of burden and stress because expenses are another concern to deal with.^47^

Costs associated to health caregiver and impact on global costs in AD have not yet been estimated.

In summary, family caregivers provide the majority of care for people with dementia, and may experience significantly higher levels of depression, anxiety and burden. Risk factors associated with increased caregiver burden include being a women caregiver, age of patient and stage of dementia.

## Economic impact of drug therapies

Economic analysis of the drug therapies in AD has been a focus of research in various studies. Analyses of intervention-based therapies show that if treatment delays institutionalization, but does not affect survival, then global costs may be lower as a result of a reduction in direct costs (cost of institutionalization). However this reduction may be attenuated by increases in indirect costs, as informal costs.^26^ In addition, if treatment prolongs survival in patients with AD, then global costs may increase because of longer assistance time.

All approved drugs in AD treatment have been subjected to economic analysis. Three cholinesterase-inhibitors, donepezil, galantamine and rivastigmine, are indicated in AD at mild and moderate stages whereas Memantine is recommended for use in moderate and severe AD.^27,28^

Feldman et al. showed that in patients with moderate-severe AD, treatment with donepezil (vs. placebo) resulted in moderately higher direct medical costs when the cost of donepezil was incorporated into the total direct medical costs. However, donepezil treatment also was associated with almost an hour per day less of caregiving time and donepezil treatment also reduced total informal costs, representing an overall net cost saving to society.^29^ These results were replicated in a recent study in Spain involving patients diagnosed with mild or moderate AD.^30^

A Galantamine study in mild or moderate AD patients reported reduced caregiver time. Caregivers in the galantamine group provided 3.5 hours less care per week (32 minutes per day) than the control group^31^ and were able to cut down on the use of costly resources such as formal home care and nursing homes, leading to cost savings over time.^32^ A study in Korea, in patients with a different socio-cultural background, showed higher direct cost in a glutamine-treated group vs. control group. However, global cost was attenuated because of reductions in caregiver burden and decreases in caregiver time.^33^

Recently, a review showed the effects of rivastigmine in improving behavioral symptoms and reducing psychotropic medication usage in nursing home residents with moderate-to-severe AD. This may also be associated with a reduction in professional caregiver burden.^34^ In turn, this may translate to lower total costs of AD. Reductions in time spent on caregiving reached 691 hours for caregivers of patients with mild AD. Treatment of patients with moderately severe AD was also evaluated but the impact was less marked, suggesting an economic benefit to early therapy.^35^ Use of rivastigmine delayed the transition to severe stages of AD and institutionalization while resulting in modest savings in direct costs of caring for patients with AD.^18^ Delays in disease progression and resulting cost savings were greater for patients who began treatment while in the milder stages of the disease. Cost savings for patients who began treatment during mild stages of AD stemmed largely from delays in transitions to moderate AD. For those who began treatment during moderate stages of AD, cost savings were mostly attributable to delayed institutionalization during the first year of treatment.^26^

Patients receiving memantine were less likely to be institutionalized and needed an average of 51.5 hours per month less caregiving time than those receiving placebo.^25^ Similarly to cholinesterase-inhibitors, direct medical costs were higher in the memantine treatment group. However, increases in direct medical costs were offset by savings in caregiving costs and direct nonmedical costs. Ultimately, total costs to society were US$ 1090 lower in the memantine group per month vs. control group. In addition, memantine increased time of independence by 40% and time to institutionalization by 15% compared to patients without the treatment.^36,37^ These studies suggested that memantine provides cost savings compared with no pharmacological treatment.

It is important to note that different methods have been used for economic analysis of drug-associated costs. In general, short-term clinical data (3-24 months) are combined with longer term projections. This may represent a limitation in analysis of costs, when assumptions are made for AD over the long term (3 to 9 years). Extensive reviews of clinical and cost-effectiveness of drug therapy in AD have been published and constitute a source of further information for readers.^39^

## Budget impact of drug treatment and comparative studies

Globally, according to a disease’s progression, costs associated to drug therapy reduce in proportion to total cost: in mild AD drugs such as cholinesterase inhibitors and other medicines represented 55.1% of total cost, 42.9% in moderate AD and 34.3% in severe AD.^5^ These observations correspond to all drugs, cholinesterase-inhibitors or memantine and drugs for concomitant medical disorders, but may reflect the lower impact of medications in more severe stages. Considering patient’s place of residence, this study showed that drug-associated cost was half in institutionalized patients (US$ 450, in previous 3 months) compared with community dwelling patients (US$ 979, in previous 3 months). Elsewhere, proportional value reduced from 56.4% at community dwelling versus 27.1% for institutionalized AD patients.

The results of a recent study showed no significant differences in annual global costs among donepezil, galantamine, and rivastigmine treatment groups: US$ 12,112 (SD 16,437), US$ 12,137 (SD 19,154) and 12,853 (SD 14,543), respectively.^38^ In this study economic analyses showed that reductions in acute health care expenditure correlate with treatment adherence regardless of chosen drug, and each additional month of cholinesterase-inhibitor treatment was associated with a 1% reduction in total all-cause health care costs (health care expenditures decreased by US$ 5.46 for every day the patient remained on therapy).

## Other cost modifiers: neuropsychiatric symptoms and co-morbidities

Neuropsychiatric symptoms are prevalent in all stages of AD and presence of these symptoms contributes toward raising all costs in AD. A large study by Herrmann et al.^21^ found that total costs multiplied when neuropsychiatric symptoms were present, evaluated with the Neuropsychiatric Inventory (NPI). It is noteworthy that no differences were found in drug-associated costs; the highest total costs for these patients were due to a greater proportion of indirect costs. There was a significant association between costs and baseline NPI. Individual symptoms such as apathy and hallucinations contributed significantly to increased costs. The authors also showed that the incremental cost for every one-point increase in NPI score was US$20 per month.

These results were confirmed in an Argentinean study recently published where behavioral symptoms, depression and functional impairment of activities of daily living increased costs in all type of dementia.^48^

## Conclusions

Consistent with the aging population seen worldwide, the global prevalence of Alzheimer’s disease is set to increase exponentially in the coming decades. Therefore, costs of AD have been subject to increasing scrutiny and projections.

The total cost of the disease, generated by the sum of direct and indirect costs, is born by relatives, other informal carers, by social health services and both public and private health systems. This total cost is influenced by many factors including the presence of neuropsychiatric symptoms, stage of disease and the drug treatment used.

The burden and time spent on caring for patients by their family members or informal caregivers increases with disease progression. This produces a high indirect cost and the full consequences of this burden have yet to be quantified (for example in increased costs due to worse health status of the caregivers). At more advanced stages of the disease, indirect costs shift over to direct costs, with the inclusion of a formal caregiver or institutionalization.

AD patients’ progression to a loss of functional capacity of the individual and the long evolution of the disease are important factors influencing the economic impact of drug treatment for the disease. The medications approved to treat Alzheimer’s disease generally increase direct medical costs. However, the impact on the total cost is beneficial since they lead to reduction of direct nonmedical and indirect costs. Delays in disease progression may also lead to partial reductions in other direct costs such as institutional costs and this “saving” in the overall cost will be greater when earlier treatment can be given.

An important feature of this review is that most of the studies examined here refer to the developed world. In developing countries, the cost dynamic or even the factors associated with caregiver burden may be different and therefore it is important to continue to investigate these and related research questions in South America.

In conclusion, AD needs to be considered when devising public health policies for the future. All aspects, including the economic and quality of life burdens for patients, relatives and health systems, should be taken into account in light of the present and future medical and socio-economic challenges associated with AD. Societies in South America will be better equipped to face these challenges if a solid body of local evidence is gathered and regularly updated.
